# Postprandial Lipemic Responses to Various Sources of Saturated and Monounsaturated Fat in Adults

**DOI:** 10.3390/nu11051089

**Published:** 2019-05-16

**Authors:** Christina M. Sciarrillo, Nicholas A. Koemel, Patrick M. Tomko, Katherine B. Bode, Sam R. Emerson

**Affiliations:** 1 Department of Nutritional Sciences, Oklahoma State University, Stillwater, OK 74078, USA; nick.koemel@okstate.edu (N.A.K.); katherine.bode@okstate.edu (K.B.B.); sam.emerson@okstate.edu (S.R.E.); 2 School of Kinesiology, Applied Health and Recreation, Oklahoma State University, Stillwater, OK 74078, USA; ptomko@okstate.edu

**Keywords:** postprandial lipemia, coconut oil, butter, canola oil, olive oil, lipid, triglycerides, dietary fat, saturated fat, cardiovascular disease

## Abstract

Background: Postprandial lipemia (PPL) is a cardiovascular disease risk factor. However, the effects of different fat sources on PPL remain unclear. We aimed to determine the postprandial response in triglycerides (TG) to four dietary fat sources in adults. Methods: Participants completed four randomized meal trials. For each meal trial, participants (n = 10; 5M/5F) consumed a high-fat meal (HFM) (13 kcal/kg; 61% of total kcal from fat) with the fat source derived from butter, coconut oil, olive oil, or canola oil. Blood was drawn hourly for 6 h post-meal to quantify PPL. Results: Two-way ANOVA of TG revealed a time effect (*p* < 0.0001), but no time–meal interaction (*p* = 0.56), or meal effect (*p* = 0.35). Meal trials did not differ with regard to TG total (*p* = 0.33) or incremental (*p* = 0.14) area-under-the-curve. When stratified by sex and the TG response was averaged across meals, two-way ANOVA revealed a time effect (*p* < 0.0001), time–group interaction (*p* = 0.0001), and group effect (*p* = 0.048), with men exhibiting a greater response than women, although this difference could be attributed to the pronounced difference in BMI between men and women within the sample. Conclusion: In our sample of young adults, postprandial TG responses to a single HFM comprised of different fat sources did not differ.

## 1. Introduction

Cardiovascular disease (CVD) is a major public health concern and the leading cause of death in the United States [1]. Traditional risk factors for CVD include smoking, physical inactivity, poor dietary habits, overweight/obesity, dyslipidemia, diabetes, and hypertension [1]. In addition, emerging evidence has given rise to consideration of postprandial changes following single, high-fat meal (HFM) consumption as substantially impacting CVD risk [2]. In fact, postprandial triglycerides (TG) have been identified as a stronger predictor of CVD risk than fasting values [2]. This is partly because individuals are in a postprandial state for the majority of their day [3]. Adverse changes that occur in the postprandial period include increases in TG [3], oxidative stress [4], inflammation [5,6], oxidized low-density lipoprotein [7], and decreases in high-density lipoprotein (HDL-C) [8,9] and vascular dilation [9], all of which have been shown to contribute to the CVD pathology.

Postprandial lipemia (PPL) is the rise in blood TG response following a meal [3]. Several studies have shown that an altered or reduced ability to clear TG in the postprandial period (thus, a large postmeal TG response) is associated with CVD [3,10,11,12]. The connection between PPL and CVD has been demonstrated in a case-control study in men with coronary heart disease (CHD) compared to healthy controls and in the sons of men with CHD compared to sons of men without CHD, where both disease-case groups exhibited significantly greater postprandial TG levels [10]. Similarly, data examined in women have revealed associations between greater postprandial TG and apolipoprotein B-48 (apoB-48) concentrations and CHD [2,12,13]. Moreover, in the Women’s Health Study and in the Copenhagen City Heart Study, both large prospective cohort studies involving women, nonfasting TG concentrations were significantly associated with increased CVD risk, even after adjustment for various confounding variables [2,14]. It has been suggested that the mechanistic connection between individual PPL and CVD is the subendothelial penetration and retention of circulating TG-rich lipoproteins (TRL) [11,15].

Given that HFMs classically used to study PPL contain >50% fat [11,16], and the variability in different dietary fat sources ability to increase or decrease CVD risk, it is logical that the source of dietary fat can modulate the postprandial TG response. Several studies have found a reduced PPL response following meals rich in monounsaturated fatty acids (MUFA) and polyunsaturated fatty acids (PUFA) compared to meals rich in saturated fatty acids (SFA) in both healthy adults and those with characteristics of metabolic syndrome [17,18,19]. These findings are in line with classical dietary data showing that certain sources of SFA are generally associated with CVD [20,21]. Meanwhile, Schwingshackl and Hoffmann [22] found that MUFA and PUFA can induce a greater PPL response compared to SFA. Given these findings and classic dietary data, the effects of different dietary fats on PPL have been inconclusive to this point. Furthermore, since the effects of a given type of dietary fat on CVD risk can also depend on the source of the fat (animal- versus plant-based SFA [23]), it is reasonable to suspect different postprandial responses based on fat source, even when those foods are comprised of similar fatty acid contents and types. In support of this concept, recent data from Teng et al. and Panth et al. examining the effects of animal- vs. plant-based SFA have yielded inconsistent and contradictory results. Teng et al. observed a lower TG response after the consumption of animal-based SFA (lard) when compared to plant-based SFA (palm olein), while Panth et al. observed a greater TG response after the consumption of animal-based SFA (butter, lard) when compared to plant-based SFA (coconut oil) [24,25]. Considering the rising scientific data on ketogenic diets and low-carb eating patterns, and their recent popularity regarding the treatment of several phenotypes associated with CVD (diabetes, obesity), it is pertinent to understand further how various sources of dietary fat affect cardiometabolic health. Furthermore, considering the inconsistency with regard to current postprandial data, and that PPL is an independent risk factor for CVD [2], determining how various types and sources of dietary fat consumed may modify individual postprandial TG response would be valuable.

Therefore, the primary purpose of this investigation was to determine the effects of commonly consumed sources of dietary fat as part of a mixed meal on PPL in young adults. Specifically, this study compared the postprandial TG response to plant-based SFA (coconut oil), animal-based SFA (butter), MUFA-rich olive oil, and MUFA-rich canola oil.

## 2. Materials and Methods

### 2.1. Participants

Ten individuals (5 M/5 W) participated in the present study and were recruited via online survey, email, or flyer from the Oklahoma State University campus. Inclusion criteria were age 18–40 years, no evidence of dietary intolerances that precluded consumption of the test meals, no chronic disease, and not taking any lipid or blood pressure medications. The study protocol was approved by the Institutional Review Board at Oklahoma State University (HE-17-77) and carried out in accordance with the Declaration of Helsinki. All participants provided verbal and written consent prior to participating in the study.

### 2.2. Overall Study Design

Participants engaged in one initial assessment and four randomized meal trials. The initial assessment consisted of detailed paperwork (informed consent, medical history questionnaire, international physical activity questionnaire (IPAQ)) and anthropometric data measurements. Participants were also administered various lifestyle control instructions during the initial assessment. Meal trials began approximately one week after the initial assessment. Each meal trial was separated by a washout period of 1–3 weeks. The sequence in which a participant consumed the four test meals was randomized. Within each meal trial, participants arrived in the laboratory 10-h fasted, a baseline blood draw was taken, they consumed the test meal, and blood draws were taken every hour for six hours post-meal to determine the postprandial TG response.

### 2.3. Initial Assessment

The initial assessment entailed detailed copies of instructions for participants to follow, completion of written informed consent, a medical history questionnaire, the IPAQ, and anthropometric evaluations. Height was measured via stadiometer (Seca 213 portable stadiometer; Seca GmbH; Hamburg, Germany). Body mass was measured using a digital scale (Seca mBCA 514; Seca GmbH; Hamburg, Germany). Blood pressure was measured using an automatic blood pressure cuff (Omron 5 Series BP742N; Omron; Kyoto, Japan). Height, weight, and blood pressure were measured twice and the average of the two measures was recorded.

Lifestyle controls were assigned and all participants were instructed to follow and comply with the explained lifestyle instructions. Lifestyle controls consisted of a three-day food record, in which participants recorded their dietary intake for the three days prior to their first meal assessment; participants then were asked to replicate their first three-day food record for the remaining three meal assessments. Accelerometers (wGT3X-BT, Actigraph; Pensacola, FL, USA) were attached to each participant’s nondominant wrist and recorded their physical activity for at least 48 h prior to each assessment. In addition, participants were asked to refrain from planned exercise for the 48 h prior to each assessment. Participants were given a 210-kcal snack, consisting of commercial peanut butter crackers (Snyder’s-Lance, Inc.; Charlotte, NC, USA), to consume the evening before each assessment, after which the 10-h fast began. Participants were given a typed copy of all detailed instructions and lifestyle controls.

### 2.4. Meal Trials

After a 10-h overnight fast, participants arrived in the laboratory on the morning of each assessment. Each meal assessment began between 6:00–8:00 A.M., depending on the scheduling availability of the participant. An indwelling 24-gauge safelet catheter (Exel International; Redondo Beach, CA, USA) was inserted into a forearm vein and a slow infusion (~1 drip/s) of 0.9% NaCl solution was initiated. Once the catheter was set, a baseline blood draw was collected. First, a 3 mL syringe (BD; Franklin Lakes, NJ, USA) was used to clear the line of saline followed by a 5 mL syringe (BD; Franklin Lakes, NJ, USA) used to take the whole blood sample. Whole blood samples were collected for the assessment of metabolic outcomes: TG, glucose, LDL-C, HDL-C, and total cholesterol (TOTAL-C). Metabolic outcomes were determined by a Cholestech LDX analyzer (Alere Inc.; Waltham, MA, USA). For each individual blood draw, a few drops of whole blood were drawn into a capillary tube and plunged into a Cholestech LDX Lipid+Glu cassette (Alere Inc.; Waltham, MA, USA). The cassette was inserted into the Cholestech LDX analyzer and processed. The CV for TG assessment via the Cholestech LDX system is approximately 2–4%. Following the baseline blood draw, participants consumed the test meal within 20 min. Water was available for participant consumption ad libitum during the meal and throughout the postprandial period. Participants remained in the laboratory for 6 h following consumption of the test meal. The 6-h time period began after the last bite of the test meal. Additional blood draws were performed every hour for the 6 h after consumption of the test meal.

### 2.5. Test Meals

The test meal consisted of pasta sauce, whole-wheat spaghetti noodles, French bread, yellow onion, green bell pepper, sea salt, black pepper, and the specific fat source being tested. Each meal contained a test fat of either MUFA-based canola oil (CaO) (Great Value, Canola Oil), MUFA-based extra virgin olive oil (OO) (Great Value, Extra Virgin Olive Oil), SFA-based virgin unrefined coconut oil (CoO) (Organic Great Value, Unrefined Virgin Coconut Oil, expeller pressed), or SFA-based grass-fed butter (B) (Kerrygold, Grass-fed Pure Irish Butter, unsalted). The test meal contained 61% of total kcal from fat, 7% of total kcal from protein, and 32% of total kcal from carbohydrate (CHO). Each participant consumed a serving of the test meal that was relative to his or her body mass (13 kcal/kg body mass). The amount of meal consumed was designed to resemble a typical serving at a restaurant or social event (1–2 servings). For each assessment, the meal was prepared independently 1–2 days prior to the assessment. To prepare each meal, the test fat was added to a small saucepan and heated over medium heat for 2 min. Onion and bell pepper were diced finely and sautéed over medium heat in a large saucepan for 3 min. The pasta sauce was added to the saucepan and brought to a boil, after which the heat was reduced, the saucepan was covered, and the mixture cooked for 7 min until the internal temperature reached 165 °F. Once the pasta sauce mixture was finished cooking, it was removed from the heat, cooled for 20 min, labeled, and stored in a BPA-free food storage container at 0 °F until needed for each assessment. The night before each assessment, the pasta sauce was thawed at 36 °F overnight. On the morning of each assessment, the noodles were prepared separately by bringing four cups of water to a boil in a medium saucepan, after which the raw noodles were added, cooked uncovered for 9 min, and strained. The pasta sauce was reheated in a small saucepan until the internal temperature reached 165 °F. The pasta sauce and noodles were combined in a small serving bowl and the French bread was served on the side. All ingredients were weighed (g) using a digital food scale (Table 1).

### 2.6. Statistical Analyses

An a priori sample size estimation, using standard deviations from previous studies [26,27], suggested that ten participants would need to be recruited to detect a clinically significant difference in the peak postprandial TG response of 0.5 mmol/L between meals with 80% power and alpha less than 0.05.

All data were assessed for normality via Shapiro–Wilk formal normality test and analysis of frequency distribution. The trapezoid method was used to calculate tAUC and incremental area under the curve (iAUC). Within each meal trial, tAUC, iAUC, peak value, and time to peak value were determined for each of the metabolic markers. These postprandial metabolic outcomes were compared across trials using a one-way analysis of variance (ANOVA) with Holm–Sidak adjustment for multiple comparisons. Time-course changes and sex-based differences in metabolic markers in the postprandial period were determined via two-way between and within (group × time) repeated measures ANOVA with a Tukey’s adjustment for multiple comparisons.

Differences between participant characteristics were compared by sex via two-tailed paired t-test. Pearson’s two-tailed correlation analysis was performed to assess the association between participant body mass index (BMI) and TG tAUC (averaged across meal trials).

A type 1 error rate of 0.05 was used in all analyses for the determination of statistically significant differences. Statistical analyses were conducted using GraphPad Prism statistical software (Version 7; GraphPad Software, Inc., La Jolla, CA, USA).

## 3. Results

### 3.1. Participant Characteristics and Premeal Physical Activity

**Figure 1:**
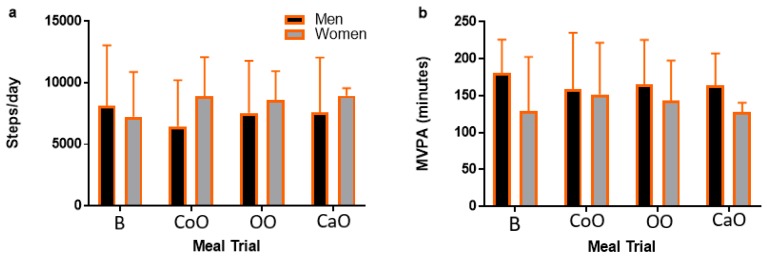
Pretrial physical activity. Physical activity in (**a**) steps/day and (**b**) MVPA for 48–72 h before each meal trial, stratified by sex. Data are presented as mean ± SD. Within each meal trial, there was no difference between men and women in physical activity in either MVPA or steps/day (*p* > 0.05). Similarly, within sex, there was no difference in physical activity across meal trials (*p* > 0.05). MVPA, moderate-vigorous physical activity; B, butter; CoO, coconut oil; OO, olive oil; CaO, canola oil.

Participant characteristics are presented in Table 2. Ten individuals participated in the present study (5 M/5 F; age: 23.8 ± 1.3 years; BMI: 25.5 ± 7.2 kg/m^2^). Based on BMI, six participants (1 M/5 F) were healthy weight (18.5–24.9 kg/m^2^), one participant (1 M) was overweight (25–29.9 kg/m^2^), and three participants (3 M) were obese (>30 kg/m^2^). One participant reported with fasting TG > 1.69 mmol/L on two occasions. Men were significantly older (mean difference: 1.2 years; *p* = 0.03) and had greater weight (mean difference: 36.2 kg; *p* = 0.01) and BMI (mean difference: 9.6 kg/m^2^; *p* = 0.02) compared to women. Men had higher fasting LDL-C concentrations when compared to women (mean difference: 0.47 mmol/L; *p* = 0.02), but there were no differences in fasting TG (*p* = 0.21), glucose (*p* = 0.96), TOTAL-C (*p* = 0.44), or HDL-C (*p* = 0.30) between men and women. Additionally, fasting TG (*p* = 0.39), glucose (*p* = 0.13), TOTAL-C (*p* = 0.07), LDL-C (*p* = 0.86), and HDL-C (*p* = 0.11) were not different across meal trials. Physical activity, measured as moderate-vigorous physical activity (MVPA) and steps/day, was not different across meal trials (*p* = 0.84 and *p* = 0.69, respectively) and there was not a main effect by meal trial (*p* = 0.69; *p* = 0.90), sex (*p* = 0.68; *p* = 0.51), or meal–sex interaction (*p* = 0.20; *p* = 0.67) (Figure 1).

### 3.2. Postprandial Metabolic Outcomes Were Similar Across Meal Trials

**Figure 2:**
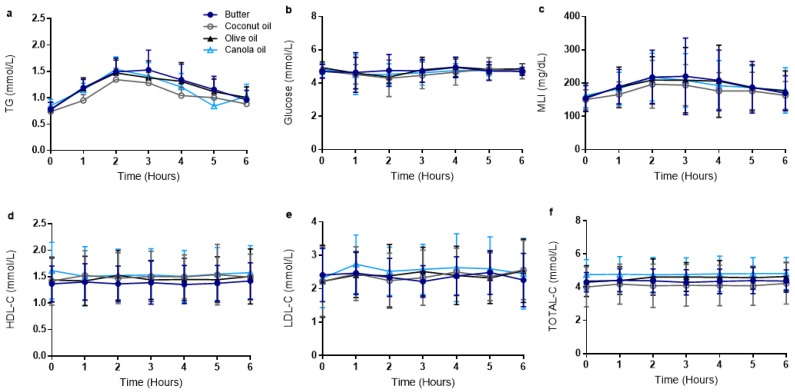
Postprandial metabolic responses. Metabolic responses in the four meal trials at baseline and hourly throughout the postprandial period for (**a**) TG, (**b**) glucose, (**c**) MLI, (**d**) HDL-C, (**e**) LDL-C, and (**f**) TOTAL-C. Data are presented as mean ± SD. Closed circles indicate B meal trial, open circles indicate CoO meal trial, closed triangles indicate OO meal trial, and open triangles indicate CaO meal trial. Error bars indicate SD. TG, triglycerides; MLI, metabolic load index; HDL-C, high-density lipoprotein cholesterol; LDL-C, low-density lipoprotein; TOTAL-C, total cholesterol.

Metabolic outcomes are presented in Table 3 and Figure 2. Two-way ANOVA of TG revealed a significant time effect (*p* < 0.0001) but no time–meal interaction (*p* = 0.56) or overall meal effect (*p* = 0.35). One-way ANOVA revealed that TG peak (*p* = 0.36) and TG time to peak (*p* = 0.23) were not different across meal trials. Meal trials did not differ with regard to TG tAUC (*p* = 0.33) or TG iAUC (*p* = 0.14). Two-way ANOVA of glucose revealed no time effect (*p* = 0.27), meal effect (*p* = 0.64), or time–meal interaction (*p* = 0.63). Glucose peak (*p* = 0.76) and glucose time to peak (*p* = 0.48) were not different across meal trials. Meal trials did not differ with regard to glucose tAUC (*p* = 0.60) or iAUC (*p* = 0.26). Two-way ANOVA of metabolic load index (MLI; calculated as TG + glucose) revealed a time effect (*p* < 0.0001) but no meal effect (*p* = 0.08) or time–meal interaction (*p* = 0.77). MLI peak (*p* = 0.24) and time to peak (*p* = 0.64) were not different across meal trials. Meal trials did not differ with regard to MLI tAUC (*p* = 0.12) or MLI iAUC (*p* = 0.08). Two-way ANOVA of LDL-C revealed no time effect (*p* = 0.27), meal effect (*p* = 0.83), or time–meal interaction (*p* = 0.72). One-way ANOVA revealed that LDL-C peak (*p* = 0.66) and time to peak (*p* = 0.59) were not different across meal trials. Meal trials did not differ with regard to LDL-C tAUC (*p* = 0.62) or iAUC (*p* = 0.72). HDL-C results did not reveal a time effect (*p* = 0.62), meal effect (*p* = 0.2), or time–meal interaction (*p* = 0.42). One-way ANOVA revealed that HDL-C peak (*p* = 0.19) and time to peak (*p* = 0.52) were not different across meal trials. Meal trials did not differ with regard to HDL-C tAUC (*p* = 0.23) or iAUC (*p* = 0.16). Two-way ANOVA of TOTAL-C revealed no time effect (*p* = 0.29), meal effect (*p* = 0.07), or time–meal interaction (*p* = 0.82). One-way ANOVA revealed that TOTAL-C peak (*p* = 0.12) and TOTAL-C time to peak (*p* = 0.09) were not different across meal trials. Meal trials did not differ with regard to TOTAL-C tAUC (*p* = 0.11) or iAUC (*p* = 0.37).

### 3.3. Postprandial Lipemic Responses Were Different between Men and Women

**Figure 3:**
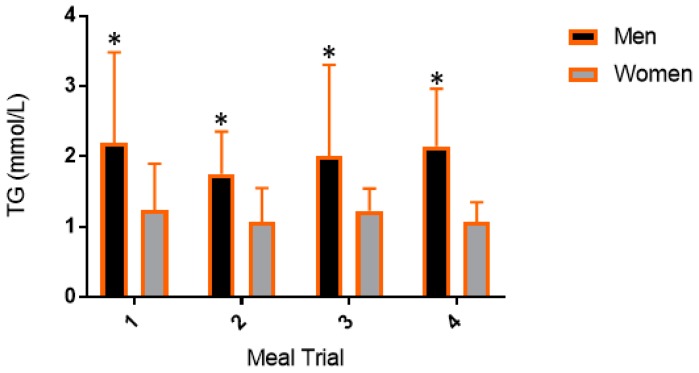
Peak response in triglycerides. Data are presented as mean ± SD. Peak TG responses for meal trials when stratified by sex. * Indicate differences between men and women for a specific meal trial (*p* < 0.05). TG, triglycerides.

When data were stratified by sex, a two-way ANOVA of TG revealed a significant time effect (men, *p* < 0.0001; women, *p* = 0.0002) but no time–meal interaction (men, *p* = 0.20; women, *p* = 0.21) or overall meal effect (men, *p* = 0.53; women, *p* = 0.48). When averaged across meal trials, men had a significantly higher TG peak (*p* = 0.03) when compared to women but there was no difference in TG time to peak between men and women (*p* = 0.87). Further, men had significantly higher TG peak (*p* < 0.05) within every meal trial (Mean sex difference: B, 1.49 mmol/L, *p* = 0.0005; CoO, 1.08 mmol/L, *p* = 0.006; OO, 1.05 mmol/L, *p* = 0.007; CaO, 1.23 mmol/L, *p* = 0.002) (Figure 3).

**Figure 4:**
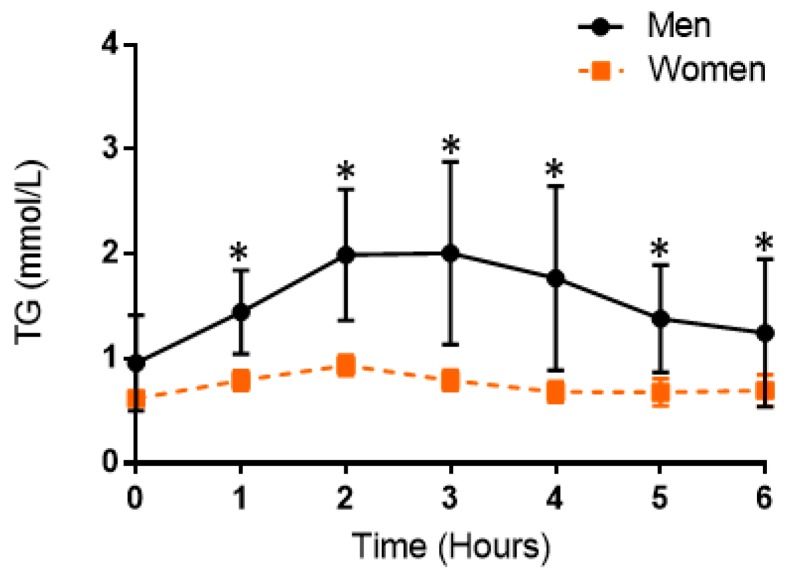
Consolidated postprandial responses in triglycerides in men and women. Average TG responses across meal trials at baseline and hourly throughout the postprandial period in men and women. Data are presented as mean ± SD. * Indicate differences between men and women at a specific time point (*p* < 0.05) based on post hoc pairwise comparison. TG, triglycerdes.

When data were stratified by sex and the TG response was averaged for each participant, a two-way ANOVA revealed a significant time effect (*p* < 0.0001), time–group interaction (*p* = 0.0001), and group effect (*p* = 0.048) (Figure 4).

**Figure 5:**
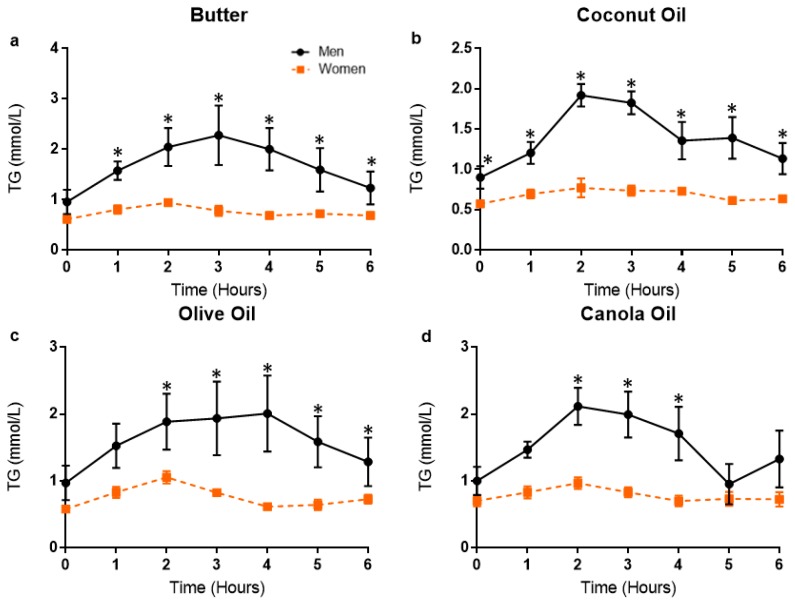
Postprandial responses in triglycerides in men and women based on meal trial. TG responses in men and women for each meal trial at baseline and hourly throughout the postprandial period. Data are presented as mean ± SD. (**a**) TG response for B meal trial; (**b**) TG response for CoO meal trial; (**c**) TG response for OO meal trial; (**d**) TG response for CaO meal trial. * Indicate differences between men and women at a specific time point (*p* < 0.05) based on *post hoc* pairwise comparison. TG, triglycerides; MLI, metabolic load index; HDL-C, high-density lipoprotein cholesterol; LDL-C, low-density lipoprotein; TOTAL-C, total cholesterol.

In post hoc pairwise testing, men had significantly higher TG than women at every time point in the postprandial period (*p* < 0.05). Postprandial TG responses in men and women within each meal trial are presented in Figure 5. When data were stratified by sex for men and women, there was a significant time effect (*p* < 0.0001, *p* = 0.0002), but no time–group interaction (*p* = 0.19, *p* = 0.21) or overall group effect (*p* = 0.53, *p* = 0.47), respectively.

**Figure 6:**
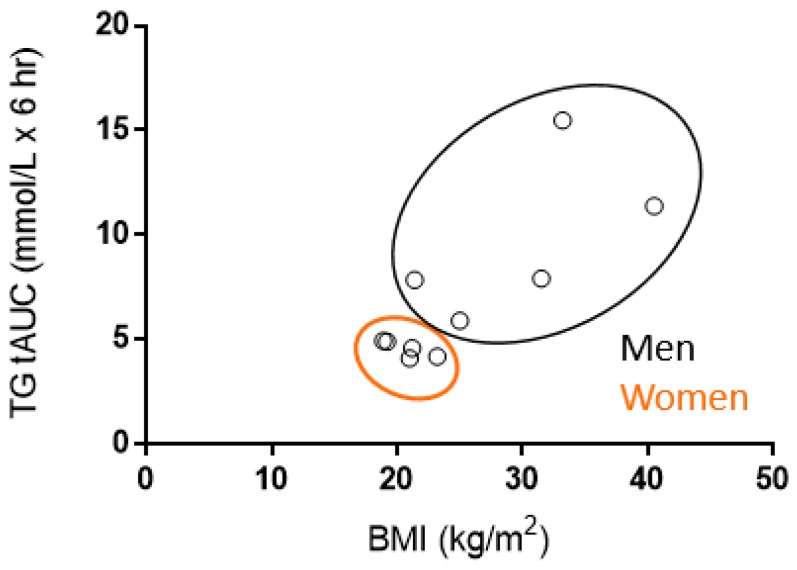
Correlation between TG tAUC and BMI. Data are means of TG tAUC for each participant (averaged across the four meal trials) and BMI (kg/m^2^) value for each individual participant. There was a significant positive correlation between BMI and TG tAUC (*r* = 0.79, *R*^2^ = 0.63, *p* = 0.006). BMI, body mass index; TG tAUC, triglycerides total area under the curve.

When data were stratified by sex for the B and OO meal trial, a two-way ANOVA of TG revealed a significant time effect (*p*’s < 0.0001) and time–group interaction (*p* = 0.001 and *p* = 0.002, respectively), but no overall group effect (*p* = 0.057 and *p* = 0.11, respectively). When data were stratified by sex for the CoO and CaO meal trial, a two-way ANOVA of TG revealed a significant time effect (*p* < 0.0001, *p* = 0.0015), time–group interaction (*p* < 0.0001, *p* = 0.04), and overall group effect (*p* = 0.02, *p* = 0.047). In post hoc pairwise testing, men had significantly higher TG at baseline, 1, 2, 3, 4, 5, and 6 h post-meal for the CoO meal trial and at 2, 3, and 4 h post-meal for the CaO meal trial. Although there was a nonsignificant group effect for the B and OO meal, in post hoc pairwise testing, men had significantly higher TG at 1, 2, 3, 4, 5, and 6 h post-meal for the B and OO meal trial. When Pearson’s two-tailed correlation was performed, BMI was strongly associated with TG tAUC (*r* = 0.79, *R*^2^ = 0.63, *p* = 0.006) (Figure 6).

## 4. Discussion

### 4.1. Postprandial Responses in Triglycerides between Meals

The present study compared the effects of a high-fat mixed meal rich in butter, coconut oil, olive oil, or canola oil on the postprandial metabolic response in young adults. Peak postprandial TG concentrations were observed at 2–4 h post-meal (mean peak across meals: 1.59 mmol/L) and suggest that the HFM used in the present study induced a robust postprandial response. In our sample of young volunteers, consumption of a mixed HFM containing various sources of commonly consumed dietary fat did not result in different postprandial TG responses. Therefore, counter to our hypotheses, the results of this study do not support the notion that various sources of dietary fat result in markedly different PPL responses. As PPL has been identified as an independent and clinically relevant risk factor for CVD, these results advance understanding with regard to the effects of different dietary fats on cardiometabolic health.

In agreement with our findings, Lesser et al. examined the lipemic effects of a mixed breakfast meal with the fat derived from almonds (MUFA) or cream cheese (dairy-based SFA) in overweight/obese pregnant women and found no significant difference in the postprandial TG response between the two meal trials (MUFA versus dairy-based SFA) [28]. Notably, the test meal utilized by Lesser et al. [28] was a mixed meal, containing a heterogeneous mixture of macro- and micronutrients. Likewise, the HFM meal used in the present study contained moderate amounts of CHO (32% of total kcal) derived from fiber-rich whole grains, French bread, and vegetables. As part of a mixed meal, fiber has been shown to blunt the PPL response by interfering with lipid absorption and digestion via impairment of proper emulsification of lipids in the gastrointestinal tract [29]. In support of this concept, Lesser et al. utilized a mixed meal consisting of 46% of calories derived from CHO and found no differences between test meals [28]. The almond test meal contained 7 g more fiber when compared to the cream cheese test meal; therefore, the lack of detectable differences between meals may have been a result of the modifying effect of fiber on PPL. Kristensen et al. found that when participants consumed a mixed meal with added fiber from flax seed, the mean TG response was 18% lower when compared to the low-fiber control, reaffirming the notion that fiber interferes with the postprandial handling of lipids [29]. Consequently, in the present study, the presence of fiber and other nutrients besides fat in the test meal may have weakened our ability to detect differences between test meals, given the buffering effect that fiber has on the magnitude of PPL.

By contrast, some previous studies have found differences in PPL based on source of dietary fat. For example, researchers examined the effects of mixed meals containing low (basmati rice) or high (jasmine rice) glycemic index CHO and three different types of dietary fat sources (B, OO, grapeseed oil) on the postprandial metabolic response in healthy adults. The TG iAUC was significantly lower following the B (SFA) and grapeseed (PUFA) meals when compared to the OO (MUFA) meal, regardless of GI [30]. These results contrast the findings of our present study that found a similar postprandial TG response when comparing the B (SFA) meal with the OO (MUFA) meal.

Similarly, Mekki et al. [26] assessed the effects of various dietary fatty acids in a mixed HFM on PPL. The authors found that, when compared to the B meal, OO induced a greater PPL response, but a comparable postprandial response to the sunflower oil meal, concluding that B resulted in lower PPL than the OO and sunflower oil meals [26]. These results contrast to our results, but align with Sun et al. [30]. Mekki et al. [26] found that the size of circulating chylomicrons (CM) were consistently lower after the meal rich in B than those detected after the meals rich in vegetable oils (OO or sunflower oil). Although not an explanation as to why these authors found differences between various dietary fats and the present study did not, the lower TG response in the B trial could have been a result of greater or faster lipolysis of CM containing fatty acids from B or a reduced overall size of secreted CM due to the calcium present in B, contributing to the formation of calcium-soap complexes. For our present study, examining the size of circulating CM and concentrations of either intestinally derived apoB-48 present in CM and/or endogenous apoB-100 present in LDL-C and very low-density lipoprotein (VLDL-C) may have yielded detectable differences between meal trials. Additionally, Mekki et al. [26] did not standardize the test meals to body weight and used a homogeneous sample consisting of only men. These factors may also partially explain the disagreement between our study and Mekki et al. [26].

In contrast to Teng et al. [24], Sun et al. [30], Mekki et al. [26], and our present study, another study observed a lower PPL response following a meal consisting of 80 g of ingested OO when compared to 100 g of ingested B [27]. However, since the OO test meal had a lower amount of total fat (80 g) compared to the B meal (100 g), these test meals were not a uniform comparison of the independent effects of OO and B on PPL. In another study investigating acute PPL [31], participants consumed either 71 g of MCT oil, representative of the predominating fatty acid found in CoO, or CaO (MUFA), and the authors found that plasma TG concentrations increased 47% from baseline after the CaO ingestion, while they increased only 15% from baseline following MCT oil ingestion. Notably, only males were included in this study sample and the test meal was not standardized to body weight, nor was it a mixed meal.

Despite several studies comparing the effects of SFA with MUFA or PUFA, there are very few examining the acute effects of various sources of SFA (plant- and animal-based) on PPL. Teng et al. [24] compared the effects of animal-based SFA (lard) and plant-based SFA (palm olein) sources to oleic MUFA-rich dietary fat (virgin OO) as part of a mixed meal on postprandial TG. Researchers found that the lard (animal-based SFA) elicited a significantly lower TG response than the OO and palm olein (plant-based SFA). On the other hand, a recent study by Panth et al. [25] examined the effects of various sources of SFA on the PPL response in healthy adults. Researchers found that the PPL response was ~60% lower after the CoO meal (plant-based SFA) when compared to the B meal (animal-based SFA) and the lard meal (animal-based SFA). No difference was observed between the B and lard meal for PPL. These findings disagree with Teng et al. [24], who found that plant-based SFA (palm olein) elicited a greater PPL response when compared to animal-based SFA (lard). Teng et al. [24] found that animal-based SFA (lard) resulted in lower postprandial TG when compared to plant-based SFA (palm olein), whereas Panth et al. [25] found that plant-based SFA resulted in lower postprandial TG when compared to two sources of animal-based SFA (butter, lard).

These findings by Teng et al. [24] and Panth et al. [25] are contradictory and there were several key differences between the two study designs. First, Teng et al. [24] recruited an exclusively male sample and employed a three-day washout period between meal trials, while Panth et al. [25] recruited equal numbers of males and females and employed a one-week washout period between meal trials. Considering the brief washout period utilized by Teng et al. [24], the effects of the dietary fat in the preceding meal trial may have carried over to the subsequent meal trial, thus influencing the postprandial response and interfering with the evaluation of a singular source of dietary fat. Additionally, Teng et al. [24] utilized a meal higher in total kcal and percent of kcal from fat (~754 total kcal; 60% total kcal from fat, 33% total kcal from CHO, 7% total kcal from protein) when compared to Panth et al. [25] (~660 total kcal; 53% total kcal fat, 40% total kcal from CHO, 5–7% total kcal from protein). Teng et al. [24] also instructed participants to abstain from consuming high-fat foods the day before the meal trials and administered a low-fat meal for the dinner preceding the day of the meal trial, while Panth et al. [25] alternatively asked participants to consume the same meal the night before each meal trial. Lastly, the postprandial assessment period employed by Teng et al. [24] consisted of BL, 1, 2, 3, and 4 h post-meal, whereas Panth [25] measured TG at BL, 2, 3, 4, and 6 h post-meal. Teng et al. [24] may not have been able to capture the entire postprandial response, considering that postprandial TG tends to peak around 2–4 h post-meal consumption and return to postabsorptive values around 6 h post-meal [2].

### 4.2. Factors Influencing the Postprandial Lipemic Response

Mixed meals contain varying amounts of macronutrients and micronutrients, which modulate physiological processes of digestion, absorption, and metabolism of fatty acids [32,33,34]. The use of laboratory-derived fat mixtures and lipid emulsions in the place of mixed meals is a common feature in studies assessing PPL (e.g., Mekki et al. [26]), particularly in those evaluating the effects of specific types of fatty acids or sources of dietary fat on PPL. Several of the studies that have observed differences in PPL based on source or type of dietary fat have utilized laboratory-derived fat mixtures or lipid emulsions [31,35,36]. Considering that individuals do not consume these dietary fat sources in isolation or as a component of lipid emulsions in daily living, testing the effects of different fats within a mixed meal may be a more practical and appropriate approach. Our study, as well as others, tested the lipemic effects of different fat sources in the context of true-to-life mixed meal and did not observe differences across meal trials. If the various dietary fat sources used in this study were isolated in laboratory-derived fat mixtures, and thus the effects of macro- and micronutrients were removed, it is possible that differences in postprandial TG between various dietary fat sources may have been observed in the present study.

We observed a strong correlation between BMI and TG tAUC. Men had significantly higher BMI than women and no females were overweight or obese. In agreement with our findings, Kasai et al. found that men with a greater BMI (≥23 kg/m^2^) compared to men with a lower BMI (<23 kg/m^2^) exhibit greater PPL in response to a HFM [37]. In contrast, Hansson et al. did not find that BMI or sex significantly altered the postprandial TG response to various types of dairy fat rich in SFA [38]. However, the study population (n = 31) consisted of 70% women and 30% men and therefore may not have been sufficient to detect an interaction between sex and postprandial TG in response to different fat sources. In addition, the median BMI was 23.6 kg/m^2^ (range: 21.0–25.8). Consequently, the range of BMI may have been too narrow to establish a relationship between BMI and postprandial TG.

The majority of studies that found various sources of dietary fat influence PPL differently included a sample of only male participants [26,30,31,33]. We also observed greater PPL responses in men for all meal trials when compared to women. This finding adds to the notion that sex is an important modifying factor with regard to PPL. There are well-known sex-based differences in visceral adipose tissue accumulation, with women generally storing less adipose tissue in the visceral region than men [39,40]. Women tend to store fat in the gynoid regions (hips/breasts/thighs), while men tend to store fat primarily in the android regions (trunk/abdomen), and thus have a tendency to accumulate fat within visceral tissues [39]. One study has suggested that this difference in visceral adipose accumulation between men and women is the primary explanation for the amplified postprandial response observed in men compared to women [39]. Additionally, Blackburn et al. found that men with impaired glucose tolerance were characterized by greater visceral adiposity, waist circumference, and postprandial lipemia when compared to men with normal glucose tolerance, adding further evidence to the notion that visceral adiposity is an important modulator of the postprandial response [41]. Further, women with android obesity, both with normal and high fasting TG, exhibit a more pronounced and deleterious postprandial TG response when compared to women with gynoid obesity with normal fasting TG [40], further supporting the influence of sex on PPL via body composition differences. Considering that men had a greater BMI than women in our sample, these findings demonstrate one possible mechanism responsible for the marked sex difference in postprandial lipemia that we observed, as the anatomical location of fat storage clearly plays a significant role in determining postprandial lipemia. Thus, although our observed sex-based differences in postprandial lipemia are noteworthy, since there were sex differences in BMI (likely indicative of differences in body composition), it is not possible to form conclusions from our study about the independent role of sex on postprandial lipemic responses.

### 4.3. Strengths and Limitations

A strength of this study was the use of a “true-to-life” mixed HFM challenge, in contrast to many studies examining PPL that use lipid emulsions or laboratory-derived lipid formulations. The meal used in the present study was also scaled to body weight and resembled a meal that individuals might typically eat at a social gathering. This consideration is important because many postprandial studies utilize meals that are unrealistically high in calories, particularly calories from fat, and are not standardized to body weight. Therefore, this study allowed for the comparison of different dietary fats with regard to PPL in a realistic context. This study also consisted of a balanced sample with regard to sex (5 M/5 F). Several studies similar in design had a predominately or exclusively male sample population. Another strength of this study was the robust postprandial assessment protocol, whereby we quantified the postprandial response serially every hour for six hours post-meal.

A limitation of this study was only measuring blood lipids and glucose. Examining the size of circulating CM and concentrations of either intestinally derived apoB-48 and/or endogenous apoB-100, in addition to blood lipids and glucose, may have been valuable with regard to answering our hypotheses. Next, all of our participants were young and presented few CVD risk factors. Thus, features of atherosclerotic development, including exaggerated and prolonged PPL, may not have been prominent enough to detect differences between meal trials, especially when considering the “true-to-life” meal used. Additionally, although this study found consistent sex-based differences in postprandial TG, it was not designed to address these differences. In addition, considering that three male participants exhibited an obese BMI (BMI > 30 kg/m^2^), it is not possible to conclude whether the greater postprandial TG response was due to sex or BMI. Similarly, the lack of body composition measurement beyond BMI was a limitation of the present study. Finally, while we conducted an a priori sample size estimation and our study featured the same sample size (n = 10) as similar previous studies [26,27], our null findings present the possibility that our study was not sufficiently powered to detect differences. A post hoc analysis revealed that, given our observed TG variations, the minimum difference in peak TG that our design could have detected was 0.54 mmol/L. Thus, while we view this to be reasonable, differences between meals less than 0.54 mmol/L could not have been statistically detected in our study.

## 5. Conclusions

In our study, the effect of various sources of dietary fat, namely plant- and animal-based SFA, on PPL did not differ. Sex-based differences regarding the PPL response to the meal trials were observed and there was a strong correlation between BMI and TG tAUC, supporting the notion that sex and BMI are important factors that modulate the acute PPL response. However, it is impossible to form conclusions about the role of sex-based differences versus differences regarding BMI within our sample, owing to the fact that BMI was different between men and women. Moreover, differences in the PPL responses to the various sources of dietary fat used in the present study were not observed, despite the inclusion of three obese individuals. It is worth noting that our null findings on the effects of different fat sources within a mixed meal on PPL were in a sample of young adults. Future studies should investigate the effects of these various dietary fat sources on PPL in populations at risk for CVD or with existing CVD. In more at-risk individuals with a larger postprandial response, differences in TG between different sources of fat may be more apparent. Overall, the magnitude of PPL in response to a realistic mixed meal is likely modulated by several interrelated dietary factors, such as the amount of fat, energy density, and the heterogeneous mixture of macro- and micronutrients, rather than the specific type or source of dietary fat alone. Future studies should continue to focus on delineating between various sources of animal-based SFA (dairy- vs. meat-based) with regard to CVD risk, both in an acute (postprandial) and chronic context.

## Figures and Tables

**Table 1:** Test fats and meal composition. Data are representative of the test meal composition for a 60 kg participant.

	Weight (g)	Energy (kcal)	Protein (g)	Fat (g)	CHO (g)	Fiber (g)
Sauce	257	577	5	52	25	3.3
Bread	28	78	3	1	16	1.5
Pasta	33	124	6	1	24	1.5
Total	318	780	14	54	65	6.3

**Table 2:** Participant characteristics. Metabolic outcomes represent fasting data averaged across the four meal trials.

	Total	Men	Women	*p*-Value
Age	23.8 ± 1.3	24.4 ± 1.5 *	23.2 ± 0.8	0.03
Weight (kg)	76.52 ± 25.23	94.57 ± 23.09 *	58.42 ± 8.79	0.01
Height (cm)	171.5 ± 10.1	176.0 ± 9.8	167.0 ± 9.1	0.10
BMI (kg/m^2^)	25.5 ± 7.2	30.3 ± 7.4 *	20.7 ± 1.7	0.02
Fasting TG (mmol/L)	0.78 ± 0.41	0.96 ± 0.53	0.61 ± 0.10	0.21
Fasting Glucose (mmol/L)	4.85 ± 0.32	4.86 ± 0.29	4.84 ± 0.38	0.96
Fasting TOTAL-C (mmol/L)	4.76 ± 0.91	5.05 ± 1.09	4.47±0.68	0.44
Fasting LDL-C (mmol/L)	2.41 ± 0.79	2.95 ± 0.42 *	1.87 ± 0.73	0.02
Fasting HDL-C (mmol/L)	1.62 ± 0.53	1.44 ± 0.68	1.79 ± 0.33	0.30
MVPA (minutes)	152.5 ± 17.3	166.9 ± 7.9	138 ± 24.4	0.51
Steps/day	7934.6 ± 747.7	7444.6 ± 1024.3	8424.6 ± 107.6	0.68

Data are presented as mean ± SD. *p*-value column indicates results of an unpaired *t*-test between men and women. * Indicates significant differences between men and women (*p* < 0.05). MVPA, moderate-vigorous physical activity; TG, triglycerides; HDL-C, high-density lipoprotein cholesterol; LDL-C, low-density lipoprotein; TOTAL-C, total cholesterol.

**Table 3:** Postprandial metabolic outcomes for the four meal trials.

	Butter	Coconut Oil	Olive Oil	Canola Oil	*p*
Triglycerides					
Peak (mmol/L)	1.7 ± 1.1	1.4 ± 0.6	1.6 ± 0.9	1.6 ± 0.8	0.36
Time to peak (hours)	2.2 ± 0.8	3.0 ± 1.5	2.8 ± 1.1	2.7 ± 1.3	0.23
tAUC (mmol/L × 6 h)	7.6 ± 4.6	6.4 ± 2.8	7.4 ± 4.7	7.1 ± 2.8	0.33
iAUC (mmol/L × 6 h)	2.9 ± 2.5	2.0 ± 1.6	2.7 ± 2.4	1.9 ± 1.7	0.14
Glucose					
Peak (mmol/L)	5.4 ± 0.9	5.3 ± 0.7	5.4 ± 0.7	5.5 ± 0.8	0.76
Time to peak (hours)	3.2 ± 1.8	2.4 ± 1.9	2.5 ± 1.8	2.5 ± 2.3	0.48
tAUC (mmol/L × 6 h)	28.5 ± 3.7	27.5 ± 3.6	28.5 ± 2.2	28.0 ± 2.8	0.60
iAUC (mmol/L × 6 h)	0.3 ± 1.5	−0.9 ± 2.3	−1.1 ± 1.1	1.1 ± 2.6	0.26
Metabolic Load Index					
Peak (mg/dL)	241.0 ± 105.4	208.8 ± 60.5	230.4 ± 99.2	231.3 ± 231.3	0.24
Time to peak (hours)	2.7 ± 1.1	2.7 ± 1.4	2.9 ± 1.2	3.2 ± 1.9	0.64
tAUC (mg/dL 6 h)	1185.2 ± 447.7	1065.5 ± 302.4	1163.7 ± 448.9	1158.9 ± 367.8	0.12
iAUC (mg/dL 6 h)	260.7 ± 239.9	163.1 ± 151.6	219.1 ± 222.1	181.9 ± 228.6	0.08
TOTAL-C					
Peak (mmol/L)	4.6 ± 0.7	4.4 ± 1.2	5.1 ± 0.9	5.0 ± 0.9	0.12
Time to peak (hours)	3.2 ± 2.3	3.6 ± 2.4	4.3 ± 1.8	2.2 ± 2.3	0.09
tAUC (mmol/L × 6 h)	26.2 ± 4.2	24.7 ± 7.3	27.3 ± 5.0	28.7 ± 5.9	0.11
iAUC (mmol/L × 6 h)	0.4 ± 1.1	0.6 ± 0.9	1.1 ± 1.5	0.2 ± 1.6	0.37
LDL-C					
Peak (mmol/L)	2.7 ± 0.6	2.8 ± 0.8	2.8 ± 0.9	2.9 ± 0.8	0.66
Time to peak (hours)	2.4 ± 2.2	3.5 ± 2.8	3.3 ± 2.5	2.6 ± 2.1	0.59
tAUC (mmol/L × 6 h)	14.2 ± 3.4	14.3 ± 4.6	14.4 ± 4.6	15.5 ± 4.9	0.62
iAUC (mmol/L × 6 h)	−0.3 ± 2.8	0.9 ± 2.7	1.1 ± 4.7	1.6 ± 5.1	0.72
HDL-C					
Peak (mmol/L)	1.5 ± 0.4	1.7 ± 0.5	1.6 ± 0.5	1.7 ± 0.6	0.19
Time to peak (hours)	3.4 ± 2.7	3.7 ± 2.0	3.5 ± 2.4	2.2 ± 2.4	0.52
tAUC (mmol/L × 6 h)	8.3 ± 2.1	8.9 ± 2.8	8.8 ± 2.5	9.2 ± 3.0	0.23
iAUC (mmol/L × 6 h)	0.1 ± 0.5	0.5 ± 0.9	0.1 ± 0.6	−0.5 ± 1.2	0.16

Data are presented as mean ± SD. There were no differences between meals for all analyses (*p* > 0.05). TG, triglycerides; MLI, metabolic load index; HDL-C, high-density lipoprotein cholesterol; LDL-C, low-density lipoprotein; TOTAL-C, total cholesterol; tAUC, total area under curve; iAUC, incremental area under the curve.
